# Supplementary material for: PRDM16 determines specification of ventricular cardiomyocytes by suppressing alternative cell fates
Source: Life Sci Alliance. 2024 Sep 20;7(12):e202402719. doi: 10.26508/lsa.202402719 (PMC11415600; doi:10.26508/lsa.202402719)

Western blot Figure S1M

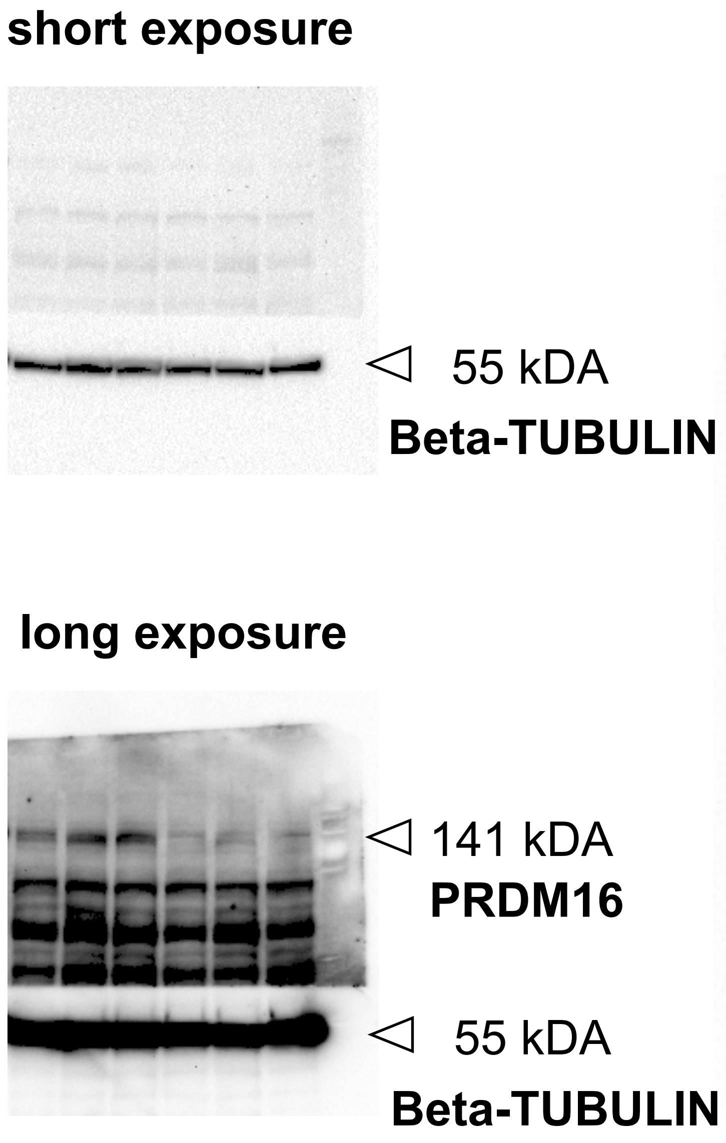

Western blot Figure S7C +D

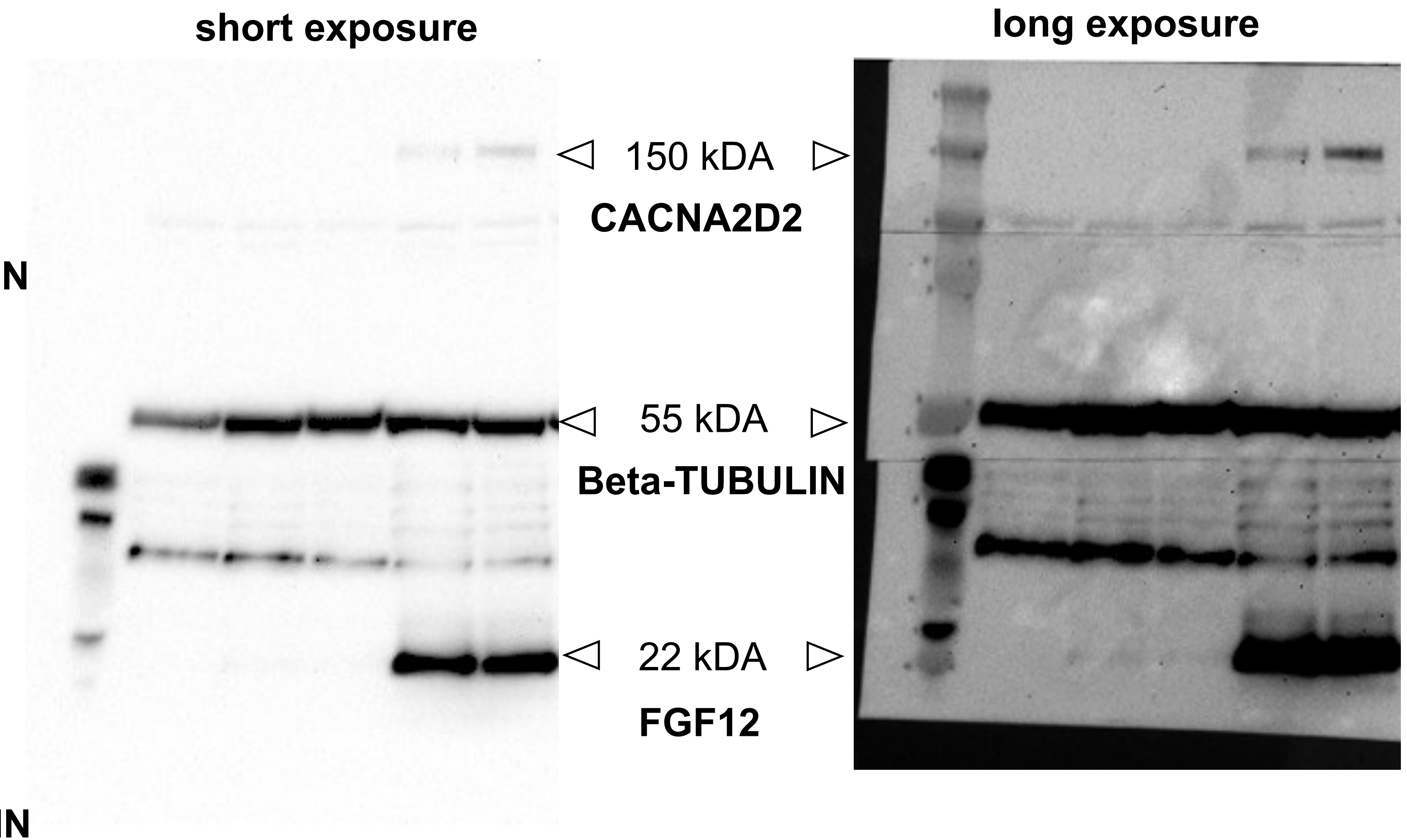

Supplement: Supplementary file 1 [file LSA-2024-02719_SdataFS1_FS7.pdf]
