# Supplementary material for: PRDM16 determines specification of ventricular cardiomyocytes by suppressing alternative cell fates
Source: Life Sci Alliance. 2024 Sep 20;7(12):e202402719. doi: 10.26508/lsa.202402719 (PMC11415600; doi:10.26508/lsa.202402719)
Supplement: Supplementary file 2 [file LSA-2024-02719_TableS1.docx]

**Table S1. Weight and echocardiography-based parameters**

| **P7** | ***Prdm16^WT^*** | ***Prdm16^cKO^*** | **P-value** | ***n*=** |
| --- | --- | --- | --- | --- |
| **BW (g)** | 4.7±0.1 | 4.1±0.2 | 0.01* | *9 vs. 8* |
| **HW/BW** | 0.007±0.0005 | 0.011±0.001 | 0.03* | *9 vs. 8* |
| **EF (%)** | 65±3 | 33±0.6 | 0.0003* | *9 vs. 9* |
| **8 weeks** | ***Prdm16^WT^*** | ***Prdm16^cKO^*** | **P-value** | ***n*=** |
| **BW (g)** | 21.6±0.7 | 22,8±0.9 | 0.27 | *9 vs. 10* |
| **HW (g)** | 0.096±0.004 | 0.104±0.003 | 0.15 |  |
| **HW/TL (g/mm)** | 0.059±0.002 | 0.064±0.002 | 0.11 |  |
| **EF male+female (%)** | 55.4±2.7 | 41.3±1.9 | 0.0004* |  |
| **EF male (%)** | 55.7±4.4** | 38.3±2.7** | 0.0044** | *4 vs. 5* |
| **EF female (%)** | 55.2±3.7** | 44.3±2.1** | 0.055 | *5 vs. 5* |
| **HR (bpm)** | 459±15 | 426±5 | 0.056 | *9 vs. 10* |
| **SV (µL)** | 30.5±1.2 | 23.3±1.9 | 0.006* |  |
| **CO (µL/min)** | 13.9±0.4 | 10.3±1.0 | 0.004* |  |
| **IVS;d (mm)** | 0.74±0.02 | 0.75±0.03 | 0.87 |  |
| **IVS;s (mm)** | 1.02±0.03 | 1.07±0.05 | 0.42 |  |
| **LVID;d (mm)** | 3.92±0.07 | 3.91±0.11 | 0.96 |  |
| **LVID;s (mm)** | 2.80±0.11 | 3.14±0.11 | 0.047* |  |
| **LVPW;d (mm)** | 0.70±0.03 | 0.68±0.05 | 0.76 |  |
| **LVPW;s (mm)** | 1.08±0.03 | 0.87±0.05 | 0.005* |  |
| **IVCT (ms)** | 13.2±0.7 | 11.5±1.1 | 0.21 |  |
| **IVRT (ms)** | 16.2±0.4 | 21.3±1.3 | 0.0018* |  |
| **MV E (mm/s)** | 735±13 | 602±32 | 0.0019* |  |
| **MV A (mm/s)** | 533±19 | 462±26 | 0.046* |  |
| **e' (mm/s)** | -34.7±2.1 | -22.5±1.5 | 0.0002* |  |
| **MV E/A** | 1.39±0.03 | 1.33±0.07 | 0.46 |  |
| **E/e'** | -21.8±1.3 | -27.4±1.5 | 0.012* |  |
| **16 weeks** | ***Prdm16^WT^*** | ***Prdm16^cKO^*** | **P-value** | ***n*=** |
| **BW (g)** | 24.7±1.5 | 24.1±1.1 | 0.69 | *5 vs. 7* |
| **HW (g)** | 0.113±0.008 | 0.15±0.02 | 0.20 |  |
| **HW/TL (g/mm)** | 0.064±0.004 | 0.074±0.005 | 0.16 |  |
| **EF male+female (%)** | 56.3±1.9 | 32.8±2.8 | <0.0001* | *13 vs. 15* |
| **EF male (%)** | 53.7±2.0** | 32.2±3.7** | <0.0001** | *8 vs. 10* |
| **EF female (%)** | 60.5±3.2** | 34.2±4.2** | 0.0003** | *5 vs. 5* |
| **HR (bpm)** | 476±17 | 426±5 | 0.056 | *13 vs. 15* |
| **SV (µL)** | 39.9±2.5 | 12.4±1.5 | 0.006* |  |
| **CO (µL/min)** | 18.7±1.0 | 10.3±1.0 | 0.004* |  |
| **IVS;d (mm)** | 0.90±0.03 | 0.89±0.04 | 0.8309 |  |
| **IVS;s (mm)** | 1.23±0.04 | 1.12±0.05 | 0.1129 |  |
| **LVID;d (mm)** | 4.11±0.09 | 4.11±0.12 | 0.9751 |  |
| **LVID;s (mm)** | 2.91±0.10 | 3.48±0.15 | 0.0048* |  |
| **LVPW;d (mm)** | 0.80±0.04 | 0.87±0.03 | 0.1831 |  |
| **LVPW;s (mm)** | 1.16±0.04 | 1.01±0.04 | 0.0154* |  |
| **IVCT (ms)** | 11.4±1.3 | 9.9±0.9 | 0.32 | *12 vs. 15* |
| **IVRT (ms)** | 14.5±1.2 | 19.5±1.2 | 0.008* |  |
| **MV E (mm/s)** | 650±17 | 617±33 | 0.4232 |  |
| **MV A (mm/s)** | 480±17 | 401±33 | 0.0579 | *11 vs. 14* |
| **e' (mm/s)** | -29.4±1.7 | -20.7±1.4 | 0.0009* | *10 vs. 10* |
| **MV E/A** | 1.36±0.05 | 1.63±0.12 | 0.059 | *11 vs. 13* |
| **E/e'** | -23.3±1.5 | -31.1±1.4 | 0.0012* | *10 vs. 10* |

*: *WT vs. cKO*, *P*<0.05 by Student’s *t*-test; **: male *vs.* female, *P*<0.05 by 2-way ANOVA with Bonferroni post-hoc test. *Abbreviations:* BW: body weight; HW: heart weight; EF: ejection fraction; HR: heart rate; SV: stroke volume; TL: tibia length; CO: cardiac output; IVS;d: interventricular septum thickness at end-diastole; IVS;s: interventricular septum thickness at end-systole; LVID;d: left ventricular internal dimension at end-diastole; LVID;s: left ventricular internal dimension at end-systole; LVPW;d: left ventricular posterior wall thickness at end-diastole; LVPW;s: left ventricular posterior wall thickness at end-systole; IVCT: isovolumic contraction time; IVRT: isovolumic relaxation time; MV E: mitral valve E-wave velocity; MV A: mitral valve A-wave velocity; E/e’: early transmitral inflow E wave over early tissue Doppler imaging e’ wave of the mitral annulus.
