# Supplementary material for: PRDM16 determines specification of ventricular cardiomyocytes by suppressing alternative cell fates
Source: Life Sci Alliance. 2024 Sep 20;7(12):e202402719. doi: 10.26508/lsa.202402719 (PMC11415600; doi:10.26508/lsa.202402719)
Supplement: Supplementary file 3 [file LSA-2024-02719_TableS2.docx]

**Table S2. Quality control of multiomics data**

|  | | ***Prdm16^WT^*** | ***Prdm16^cKO^*** |
| --- | --- | --- | --- |
| **Number of nuclei** | *After CellBender* | **4,248** | **3,202** |
| *ATAC median high-quality*  *fragments per cell* | | 10,525 | 11,027 |
| *RNA medium UMI*  *counts per cell* | | 5,774 | 4,670 |
| *Median genes per cell* | | 2,419 | 2,227 |
| **Number of nuclei** | *After DblFinder* | **3,795** | **2,784** |
| **Final number of nuclei** | *After General QC* | **3,192** | **2,288** |
| **General QC parameters** | | ***Prdm16^WT^*** | ***Prdm16^cKO^*** |
| nFeature_RNA | | 400<X<6,000 | 400<X<6,000 |
| % mitochondrial RNA | | <5 | <5 |
| nCount_RNA | | >600 | >400 |
| log10Genes per UMI | | >0.8 | >0.8 |
| nCount_ATAC | | 500<X<100,000 | 500<X<100,000 |
| nucleosome_signal | | <2 | <2 |
| TSS-enrichment | | >2 | >2 |

*Abbreviations*: QC: quality control; TSS: transcriptional start site; UMI: unique molecular identifier.
